# Multitrack Compressed Sensing for Faster Hyperspectral Imaging

**DOI:** 10.3390/s21155034

**Published:** 2021-07-24

**Authors:** Sharvaj Kubal, Elizabeth Lee, Chor Yong Tay, Derrick Yong

**Affiliations:** 1 Singapore Institute of Manufacturing Technology, Agency for Science, Technology and Research, 2 Fusionopolis Way, Singapore 138634, Singapore; sharvaj.kubal@smart.mit.edu; 2 Critical Analytics for Manufacturing Personalized-Medicine, Singapore-MIT Alliance for Research and Technology Centre, 1 Create Way, Singapore 138602, Singapore; elizabeth.lee@smart.mit.edu; 3 School of Materials Science and Engineering, Nanyang Technological University, 50 Nanyang Ave., Singapore 639798, Singapore; cytay@ntu.edu.sg; 4 School of Biological Sciences, Nanyang Technological University, 50 Nanyang Ave., Singapore 639798, Singapore

**Keywords:** hyperspectral imaging, compressed sensing, wavelets, adaptive imaging

## Abstract

Hyperspectral imaging (HSI) provides additional information compared to regular color imaging, making it valuable in areas such as biomedicine, materials inspection and food safety. However, HSI is challenging because of the large amount of data and long measurement times involved. Compressed sensing (CS) approaches to HSI address this, albeit subject to tradeoffs between image reconstruction accuracy, time and generalizability to different types of scenes. Here, we develop improved CS approaches for HSI, based on parallelized multitrack acquisition of multiple spectra per shot. The multitrack architecture can be paired up with either of the two compatible CS algorithms developed here: (1) a sparse recovery algorithm based on block compressed sensing and (2) an adaptive CS algorithm based on sampling in the wavelet domain. As a result, the measurement speed can be drastically increased while maintaining reconstruction speed and accuracy. The methods were validated computationally both in noiseless as well as noisy simulated measurements. Multitrack adaptive CS has a ∼10 times shorter measurement plus reconstruction time as compared to full sampling HSI without compromising reconstruction accuracy across the sample images tested. Multitrack non-adaptive CS (sparse recovery) is most robust against Poisson noise at the expense of longer reconstruction times.

## 1. Introduction

Hyperspectral images are comprised of light intensity information from an object or scene, resolved into two spatial dimensions and a spectral (wavelength) dimension, forming a datacube. Such images can be interpreted as a collection of (i) electromagnetic spectra associated with each spatial pixel or (ii) grayscale images corresponding to each of the spectral channels.

Hyperspectral imaging (HSI)—the capture of hyperspectral images—proves beneficial in areas as diverse as biomedicine [1], materials defect detection [2], agriculture monitoring and food safety [3], and even beyond more traditional applications, such as remote sensing of terrain [4]. Variants of HSI, such as Raman chemical imaging [5], where each spatial pixel records a Raman spectrum, are useful as label-free compositional analysis methods for spatially heterogeneous samples. Such tools are valuable in biopharmaceutical and cell manufacturing.

The biopharmaceutical industry has been trending towards integrated continuous manufacturing [6], where real-time measurements and process control [7] are critical in ensuring and optimizing the quality of the products. This is particularly important due to the stringent regulatory landscape. Here, optical techniques such as HSI can provide information-rich yet non-contact methods for monitoring and inspection.

Hyperspectral imaging presents a challenge due to the large amount of data that has to be acquired (and subsequently stored) and the long duration of time required to acquire this data. Established methods that fully sample the hyperspectral scene include point-scan, line-scan (pushbroom), wavelength-scan and snapshot imaging. (For clarity in description, we distinguish between the terms hyperspectral *scene* and hyperspectral *image*. The former will refer to the object or scene that is *to be imaged* hyperspectrally; the latter will refer to the datacube captured by an imager.) Scanning methods can be time-consuming, especially in low-light conditions where long integration times are needed. These methods also often involve moving parts. Furthermore, if the scene to be imaged is dynamic, scanning can produce artifacts that are difficult to correct during postprocessing. On the other hand, snapshot imaging methods, which record the whole image in one shot, involve very large detector arrays and complex optical arrangements such as lenslet arrays and filter arrays [8]. Overall, there is a necessity for subsampling (or undersampling) approaches to HSI such that measurements can be made faster and more efficient without losing critical information in the process.

A standard approach to the subsampling of signals is compressed sensing (CS), also known as compressive sensing or compressive sampling [9,10]. The theory of compressed sensing prescribes a subsampling procedure, together with a reconstruction algorithm, which recovers the signal (the datacube in our case) by making use of some prior assumptions about the structure of the signal. Recent HSI approaches have been leveraging on this theory in a variety of ways [11]. Coded aperture snapshot spectral imaging (CASSI) [12,13,14] provides a prominent class of imaging architectures for compressed sensing HSI (CS-HSI), that can achieve a snapshot measurement—capturing all the necessary data in one shot instead of using a sequence of measurements. Another important HSI architecture is the single pixel camera (SPC)-style hyperspectral imager [15], which is a direct extension of the SPC by replacing the single pixel detector with a spectrometer. There have also been recent efforts in making CS-based hyperspectral imagers more compact. The use of spatial light modulators or coded apertures is forgone in [16,17], and the number of optical elements required is greatly reduced.

Classes of CS algorithms commonly employed are sparse recovery [18,19] (which can be augmented by dictionary learning [20] or learnt nonlinear representations [21]), neural network-based reconstruction [22,23,24,25] and adaptive basis scan (or adaptive direct sampling) [26,27,28,29]. Sparse recovery algorithms tend to have good performance guarantees but are generally computationally intensive, making them slow for large signals such as hyperspectral scenes. Neural network-based reconstruction, on the other hand, is much faster. Neural networks use training signals to learn an inverse transformation from the subsampled measurements back to the original signal, hence avoiding slow optimization steps post-training. However, a lack of sufficient training data or mismatches between the distributions of the training set and the set of target signals can limit the generalizability and scope of this method. Lastly, an adaptive basis scan is based on progressive prediction and sampling of the most significant coefficients of a signal in some basis. These prediction steps are computationally lighter than the iterative steps involved in sparse recovery, and as a result, adaptive basis scan also achieves fast reconstruction speeds. Moreover, it is not limited by the need for large training datasets, unlike neural-network-based methods.

In the context of contemporary manufacturing, where there is an increasing need for high-volume production with extensive personalization [30], we require responsive yet autonomous operations together with end-to-end integration of the various processes. The monitoring and inspection systems involved in such a setting would require real-time processing and feedback while maintaining reliability, robustness and consistency [31].

CASSI is not very suitable for these purposes since it cannot be used with an adaptive basis scan (to our knowledge); it fails the ‘fast reconstruction’ criterion when using sparse recovery and the ‘generalizability’ criterion when using neural-networks for reconstruction. The alternative here, the SPC-style hyperspectral imager, is also limited in its capability as it only measures one spectrum in a shot. Hence, a large number of shots are needed to obtain sufficient information for a good reconstruction of the hyperspectral datacube. As a consequence, the measurement times are high despite the fact that reconstruction times can be reduced by computationally light algorithms. Overall, there is a need for HSI methods that can achieve fast measurement as well as reconstruction while maintaining imaging accuracy and generalizability across various types of scenes.

Here, we formulate improved compressed sensing approaches for HSI, which overcome the aforementioned limitations of measurement and reconstruction speeds. This is achieved by multitrack compressed sensing, based on a multitrack acquisition architecture enabling multiple spectra to be measured in each shot. Besides being compatible with standard sparse recovery techniques, multitrack compressed sensing also supports an adaptive basis scan so that multiple basis coefficients can be sampled in parallel. As a result, the measurement speed can be drastically improved while maintaining reconstruction speed as well as accuracy.

The paper is organized as follows. Section 2 reviews the prior work on compressed sensing for HSI, describes the proposed multitrack acquisition architecture and develops the two compatible CS algorithms for it: a non-adaptive sparse-recovery-based algorithm and an adaptive basis scan algorithm. Section 3 describes the numerical experiments conducted and discusses the reconstruction results for noiseless as well as noisy measurements. Finally, Section 4 concludes.

## 2. Materials and Methods

### 2.1. Background

#### 2.1.1. CS Formulation and Notation

Here, we describe the general framework for compressed sensing [9,10]. Let $x\in R^{n}$ (represented by a column vector) be a discrete signal that is to be measured. The measurement (alternatively sampling or acquisition) of *x* is described by the equation:

(1) y=Φx

where $y\in R^{m}$ is a column vector whose components are all the measurement values, and $\Phi \in R^{m\times n}$ is the measurement matrix. An individual measurement $y_{j}$ can be written as a dot product (or projection) $y_{j}=\varphi_{j}^{T}x$ between the signal and the *j*th row $\varphi_{j}^{T}$ of Φ. Subsampling occurs when the number of measurements *m* is less than the length of the signal *n*, i.e., when the sampling ratio $r_{\mathrm{sampling}}=m/n$ is less than 1. After the subsampling process, the original signal *x* is reconstructed from the measurements *y* by methods such as sparse recovery.

The particular type of signals addressed in this paper is hyperspectral scenes (and images), which can be naturally organized into datacubes. Mathematically, this is a 3D array of the shape $n_{h}\times n_{w}\times n_{c}$, where $n_{h}$ is the spatial height of the scene or image in pixels, $n_{w}$ is the spatial width in pixels, and $n_{c}$ is the number of wavelength channels. $n_{p}\equiv n_{h}\;n_{w}$ denotes the total number of spatial pixels. It is common and convenient for use in later equations, however, to reshape the elements of the datacube into a matrix: $X\in R^{\left(n_{h}n_{w}\right)\times n_{c}}$, whose row indices correspond to spatial pixel locations and column indices correspond to wavelength channels. This is performed by taking spatial slices of the datacube at each wavelength channel and unrolling these slices into tall column vectors, which end up forming the columns of *X*.

Note that the symbol *X* here plays the same role as the symbol *x* in Equation (1)—the only difference is that the elements of *X* are arranged into a matrix instead of a column vector. This notation will be used in the following sections of the paper.

#### 2.1.2. Acquisition Architectures

Several CS acquisition architectures that have been employed [11] make use of devices that can modulate and code light spatially. Spatial light modulators include masks [32], which block out light in some regions and not in others, and digital micromirror devices (DMD) [33], which are arrays of micrometer-scale mirrors that can dynamically form patterns that selectively redirect light from different regions.

Coded aperture snapshot spectral imaging (CASSI) [12,13,14] provides a prominent class of imaging architectures for CS-HSI, which can achieve snapshot HSI. The snapshot nature enables capturing hyperspectral video at high frame rates [34]. However, such powerful architectures can come with increased hardware costs—to image an $n_{h}$(height)-by-$n_{w}$(width)-by-$n_{c}$(wavelength channels) voxel scene using single dispersive CASSI, for example, the detector array needs to have dimensions of at least $\left(n_{h}\right)\times \left(n_{w}+n_{c}-1\right)$ pixels. The number $n_{w}+n_{c}-1$ can generally be very large, and this additionally presents a trade-off between the amount of spatial and spectral information that can be captured at once. Dual dispersive CASSI can function with a detector array having fewer pixels, but there is still the issue that significantly low amounts of spectral information can be captured in one shot. Multiframe CASSI [35] acquires more information through multiple shots, each using a distinct mask or DMD pattern, albeit the snapshot nature of the method is inevitably lost. Hence, there exists a different trade-off: one between spectral information and acquisition time. Furthermore, CASSI systems use prisms as the dispersive element and since the dispersion of a prism is nonlinear, the spectral resolution of CASSI varies with the wavelength [36].

High-accuracy and high-resolution spectral data warrant the use of a spectrometer. The single pixel camera (SPC)-style hyperspectral imager [15] is an acquisition architecture that leverages this—it consists of an SPC with the single pixel detector replaced by a spectrometer. Being an extension of the SPC, this architecture allows us to borrow many of the CS methods developed originally for single pixel imaging, including an adaptive basis scan. The capability of the architecture to sample dot products of patterns on the DMD with the spatial slices of the datacube allows for direct sampling of the wavelet coefficients of the scene. Note, however, that unlike snapshot CASSI, the SPC-style imager requires sequential measurements of spectra through a large number of shots. Therefore, even though faster adaptive reconstruction techniques are enabled, the slow measurement process nullifies this advantage. Addressing this limitation is the central theme of this work.

#### 2.1.3. Adaptive Basis Scan Algorithms

Adaptive basis scan was briefly described in Section 1. It is based on progressive prediction and sampling of the most significant coefficients of signals in some basis. The most popular bases for this method are wavelets [37]. Wavelet transforms decompose signals into coefficients that capture the signals’ features at multiple scales and locations. Such multiresolution analyses provide the transform coefficients with a hierarchical structure so that some coefficients have predictive power over others. In particular, low-resolution information that is contained in the wavelet coefficients at coarser scales can be used to predict which of the wavelet coefficients at finer scales are significant.

Deutsch et al. [26] predicted the significance of the fine-scale coefficients by estimating local directional Lipschitz exponents from the already-sampled coarse-scale coefficients. Averbuch et al. [27], on the other hand, relied on statistical modeling of the wavelet coefficients and their parent–child relationships. In [38], Hahn et al. adapted Deutsch’s adaptive algorithm to hyperspectral imaging. Finally, Rousset et al. [29] developed an adaptive basis scan algorithm whose wavelet coefficient prediction step relies on bicubic interpolation in image space. This was further extended to time-resolved HSI in [39]. In [38,39], however, the measurement step is not multitrack—the sensor elements measure only a single spectrum per shot.

The multitrack adaptive CS algorithm we present in Section 2.2.3 is an extension of the Adaptive Basis Scan with Wavelet Prediction (ABS-WP) algorithm from [29,39]. We make this choice because ABS-WP produces high accuracy reconstruction, even in the presence of noise. Furthermore, ABS-WP has already been demonstrated to work with HSI via minor changes to the original algorithm. However, multitrack CS is potentially compatible with the other adaptive basis scan algorithms as well.

### 2.2. Proposed Multitrack Compressed Sensing

The single-track acquisition of the SPC-style hyperspectral imager measures only one spectrum per shot, and hence, a large number of shots are needed to obtain sufficient information for a good reconstruction of the hyperspectral scene. This is especially a problem when integration times are large, as is the case for low-light imaging. To develop more powerful acquisition architectures, one can draw inspiration from pushbroom hyperspectral imagers in which multiple spectra are measured at once.

#### 2.2.1. Multitrack Acquisition Architecture

**Figure 1:**
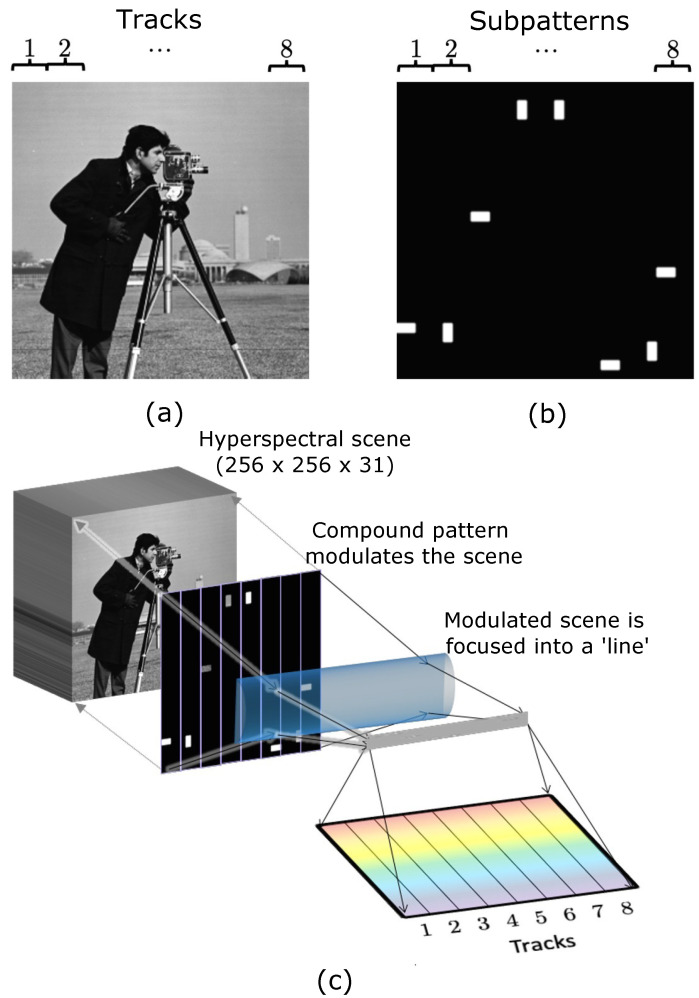
(**a**) Dividing a hyperspectral scene into tracks (8 tracks as an example) seen here as the dashed vertical strips. (**b**) A full compound pattern as formed by subpatterns (8 subpatterns in this example), again seen as the dashed vertical strips. (**c**) A schematic of the multitrack acquisition process. In every shot, the hyperspectral scene is modulated by a compound pattern, focused into a line and dispersed in a spectrometer. The 2D detector array records one spectrum from each track.

Consider the hyperspectral scene $X\in R^{n_{p}\times n_{c}}$, whose components have units of photons per second. Different spatial regions—rectangular tracks (Figure 1a)—of the hyperspectral scene are to be treated separately yet in parallel. Let $n_{\tau }$ be the number of tracks. Then, each track of the scene will be $n_{h}$-by-$\left(n_{w}/n_{\tau }\right)$-by-$n_{c}$ in terms of voxels. Spatially, each track has $n_{h}\times \left(n_{w}/n_{\tau }\right)=n_{p}/n_{\tau }$ pixels. In our matrix notation of *X*, this division into tracks can be described by breaking down *X* into submatrices:

(2) $$X=\left[\begin{array}{c} X^{\left(1\right)}\\ \vdots \\ X^{\left(n_{\tau }\right)} \end{array}\right]$$

where $X^{\left(1\right)},\ldots ,X^{\left(n_{\tau }\right)}\in R^{\left(n_{p}/n_{\tau }\right)\times n_{c}}$ represent the tracks. Next, the different tracks of *X* need to be modulated separately but in parallel. This is achieved by dividing the DMD into tracks of size $n_{h}$-by-$\left(n_{w}/n_{\tau }\right)$ as well to correspond spatially to the tracks of the hyperspectral scene (Figure 1b). The pixels on the *i*th track of the DMD form the *i*th subpattern $\zeta^{\left(i\right)}\in R^{\left(n_{p}/n_{\tau }\right)}$. This modulates the *i*th track of *X*, i.e., $X^{\left(i\right)}$. All tracks of *X* simultaneously undergo such modulation, after which the entire modulated scene is focused into a line by a cylindrical lens (Figure 1c). Different sections of the line are comprised of information from different tracks. This can be modeled by a trackwise list of dot products between subpatterns and the corresponding tracks of *X*:

(3) $$\left[\begin{array}{c} {\left(\zeta^{\left(1\right)}\right)}^{T}X^{\left(1\right)}\\ \vdots \\ {\left(\zeta^{\left(n_{\tau }\right)}\right)}^{T}X^{\left(n_{\tau }\right)} \end{array}\right]\in R^{n_{\tau }\times n_{c}}$$

where each ${\left(\zeta^{\left(i\right)}\right)}^{T}X^{\left(i\right)}\in R^{1\times n_{c}}$. Finally, the transformed scene enters a spectrometer where the focused line is dispersed. The 2D detector array captures one spectrum from each track, i.e., $n_{\tau }$ spectra in total. With integration time Δt, the recorded spectra in a given shot (in the units of photon counts) are described by the rows of:

(4) $$\left[\begin{array}{c} {\left(\zeta^{\left(1\right)}\right)}^{T}X^{\left(1\right)}\Delta t\\ \vdots \\ {\left(\zeta^{\left(n_{\tau }\right)}\right)}^{T}X^{\left(n_{\tau }\right)}\Delta t \end{array}\right]\in R^{n_{\tau }\times n_{c}}$$

assuming no measurement noise. Overall, one shot of the multitrack acquisition process comprises of the simultaneous (or parallel) action of subpatterns $\zeta^{\left(1\right)},\ldots ,\zeta^{\left(n_{\tau }\right)}$ to linearly map *X* onto the recorded spectra ${\left(\zeta^{\left(i\right)}\right)}^{T}X^{\left(i\right)}\Delta t$.

#### 2.2.2. Multitrack Non-Adaptive Compressed Sensing

This section describes the first of our proposed methods. It is based upon block compressed sensing and uses non-adaptive sparse recovery for reconstruction. Block compressed sensing [40,41] involves dividing a scene into blocks and applying the measurement matrix to those blocks separately. The reconstruction can then be carried out either separately for each block or globally by processing all the blocks together. The aim of block CS is to reduce the reconstruction complexity and the memory burden of the imaging process.

The track-wise acquisition naturally allows for the use of block compressed sensing methods, where the tracks can be treated as blocks that have their own measurement matrices. The reconstruction algorithm then takes into account this measurement matrix structure, as will be described later.

Note that a non-adaptive compressed sensing HSI architecture with a similar multitrack acquisition principle was first developed, to our knowledge, in [42]. Per shot, a spectrum is captured from each pixel-wide column of the scene; this is the same as having one-pixel-wide tracks in our formulation from Section 2.2.1. The use of block compressed sensing provides a generalization to this, allowing the tracks to have a width greater than one pixel. This is important when the number of spectra that can be measured per shot is limited by the detector pixel count. Several spectrometers have 2D detector arrays of 256-by-1024 pixels, for example, where a 1-by-1024 strip is meant for a single spectrum. In that case, the maximum number of spectra that can be measured in a shot is 256. Moreover, due to optical aberrations near the edges of the detector array, the maximum number of spectra might be even lower in practice. This is a limiting factor when the desired datacube is more than 256 pixels wide. Our use of block CS, however, enables the use of wider tracks. As a result, fewer tracks can be used cover the whole datacube, and fewer spectra need to be measured per shot.

First, we describe the measurement process. As separate tracks are measured separately, we can rewrite Equation (1) for each track:

(5) $$Y^{\left(i\right)}=\Phi^{\left(i\right)}X^{\left(i\right)}\;\;\;\mathrm{for}\mathrm{each}\mathrm{track}i$$

where $\Phi^{\left(i\right)}\in R^{m^{\left(i\right)}\times \left(n_{p}/n_{\tau }\right)}$ is track *i*’s measurement matrix, and $m^{\left(i\right)}$ is the number of samples taken from track *i*, which is related to the sampling ratio by $m^{\left(i\right)}=r_{\mathrm{sampling}}\times n_{p}/n_{\tau }$. $Y^{\left(i\right)}\in R^{m^{\left(i\right)}\times n_{c}}$ are the measured spectra from the track (up to some scaling factors). The measurement matrices $\Phi^{\left(i\right)}$ we use are composed of entries drawn from a transformed Bernoulli distribution: $\pm \;1/\sqrt{m^{\left(i\right)}}$ with equal probabilities. Here $\sqrt{m^{\left(i\right)}}$ works as a normalization.

Now, we describe how the measurement matrices can be implemented using a DMD. Consider track *i*. The *k*th projection from this track can be written as the dot product

(6) $$Y_{k,\cdot }^{\left(i\right)}={\left(\varphi_{k}^{\left(i\right)}\right)}^{T}X^{\left(i\right)}$$

where $Y_{k,\cdot }^{\left(i\right)}$ denotes the *k*th row of $Y^{\left(i\right)}$ (equivalent MATLAB-like notation would be $Y^{\left(i\right)}\left(k,:\right)$), and ${\left(\varphi_{k}^{\left(i\right)}\right)}^{T}$ is the *k*th row of $\Phi^{\left(i\right)}$. To implement this projection, we employ the following scaling and shifting operations on $\varphi_{k}^{\left(i\right)}$ and obtain physically implementable *b*-bit (grayscale) subpatterns:

(7) $$\zeta_{k}^{\left(i\right)}=\frac{2^{b-1}-1}{2^{b}-1}\left(\frac{\varphi_{k}^{\left(i\right)}}{{\left\|\varphi_{k}^{\left(i\right)}\right\|}_{\infty }}+1_{n_{p}}^{\left(i\right)}\right)\;\;\;\mathrm{for}\mathrm{all}\mathrm{tracks}i$$

Here, ${\left\|\cdot \right\|}_{\infty }$ denotes the $\ell_{\infty }$-norm, and $1_{n_{p}}^{\left(i\right)}$ is a subpattern ${\left[1,\ldots ,1\right]}^{T}\in R^{\left(n_{p}/n_{\tau }\right)}$ of the all-ones pattern $1_{n_{p}}\in R^{n_{p}}$:

$$1_{n_{p}}=\left[\begin{array}{c} 1_{n_{p}}^{\left(1\right)}\\ \vdots \\ 1_{n_{p}}^{\left(n_{\tau }\right)} \end{array}\right]$$

An additional offset subpattern is also required to compensate for the scaling and shifting mentioned above:

(8) $$\zeta_{\mathrm{offset}}^{\left(i\right)}=\frac{2^{b-1}-1}{2^{b}-1}\;1_{n_{p}}^{\left(i\right)}\;\;\;\mathrm{for}\mathrm{all}\mathrm{tracks}i$$

These operations are needed because $\varphi_{k}^{\left(i\right)}$ cannot be physically implemented in a direct manner. As the DMD pixels values are physically restricted to the interval [0,1], the $\pm 1/\sqrt{m^{\left(i\right)}}$ values of $\varphi_{k}^{\left(i\right)}$ cannot be realized on the DMD. Furthermore, *b*-bit DMD pixel values are subject to the discretization $\left\{\frac{0}{2^{b}-1},\frac{1}{2^{b}-1},\ldots ,\frac{2^{b}-1}{2^{b}-1}\right\}$, which needs to be accounted for while constructing the subpatterns.

Next, these subpatterns are formed on the DMD to obtain raw measurements parallel to each track. For the *k*th shot, we get:

(9) $$G\left(k\right)=\left[\begin{array}{c} {\left(\zeta_{k}^{\left(1\right)}\right)}^{T}X^{\left(1\right)}\Delta t\\ \vdots \\ {\left(\zeta_{k}^{\left(n_{\tau }\right)}\right)}^{T}X^{\left(n_{\tau }\right)}\Delta t \end{array}\right]$$

with integration time Δt. From the offset subpatterns, we obtain

(10) $$G_{\mathrm{offset}}=\left[\begin{array}{c} {\left(\zeta_{\mathrm{offset}}^{\left(1\right)}\right)}^{T}X^{\left(1\right)}\Delta t\\ \vdots \\ {\left(\zeta_{\mathrm{offset}}^{\left(n_{\tau }\right)}\right)}^{T}X^{\left(n_{\tau }\right)}\Delta t \end{array}\right]$$

The raw measurement can be processed as follows, based on Equations (6)–(10) to obtain the elements of *Y*:

(11) $$Y_{k,\ell }^{\left(i\right)}=\frac{2^{b}-1}{2^{b-1}-1}\frac{{\left\|\varphi_{k}^{\left(i\right)}\right\|}_{\infty }}{\Delta t}\left({\left[G\left(k\right)\right]}_{i,\ell }-{\left[G_{\mathrm{offset}}\right]}_{i,\ell }\right),\;\;\mathrm{for}\mathrm{all}i,\ell$$

where ${\left[G\left(k\right)\right]}_{i,\ell }$ and ${\left[G_{\mathrm{offset}}\right]}_{i,\ell }$ denote (i,ℓ)th entries of G(k) and $G_{\mathrm{offset}}$, respectively. Note that $G_{\mathrm{offset}}$ does not depend on the projection index *k*, and thus, it only needs to be measured once. The measured value can subsequently be reused for other values of *k*. However, in the case of noisy measurements, it would be preferable to measure $G_{\mathrm{offset}}$ a few more times, with an average taken in the end.

Now, we describe the reconstruction algorithm based on block compressed sensing.

A direct way to approach reconstruction would be to use sparse recovery on each track separately. However, this leads to blocking artifacts. An alternative solution for block compressed sensing is to reformulate Equation (5) as

(12) $$\underset{Y}{\underbrace{\left[\begin{array}{c} Y^{\left(1\right)}\\ \vdots \\ Y^{\left(n_{\tau }\right)} \end{array}\right]}}=\underset{\Phi }{\underbrace{\left[\begin{array}{ccc} \Phi^{\left(1\right)}\\ \; & \ddots \\ \; & \; & \Phi^{\left(n_{\tau }\right)} \end{array}\right]}}\underset{X}{\underbrace{\left[\begin{array}{c} X^{\left(1\right)}\\ \vdots \\ X^{\left(n_{\tau }\right)} \end{array}\right]}}$$

Sparse recovery can now be used to solve for all of *X* from the measurements *Y*. Though *X* can now be reconstructed globally, it is often simpler and more efficient to reconstruct spatial slices $X_{\cdot ,\ell }$ for the different wavelength channels $\ell \in \{1,\ldots ,n_{c}\}$ separately. Note that $X_{\cdot ,\ell }$ denotes the *ℓ*th column of *X* (equivalent MATLAB-like notation would be X(:,ℓ)). This yields

(13) $$Y_{\cdot ,\ell }=\Phi X_{\cdot ,\ell }\;\;\;\mathrm{for}\mathrm{each}\mathrm{channel}\ell$$

where $Y_{\cdot ,\ell }$—the *ℓ*th column of *Y*—are the measurements at the *ℓ*th wavelength channel. The reconstruction algorithm we employ is the 2D total variation (TV)-minimization (used on each slice separately):

(14) $$\hat{X_{\cdot ,\ell }}=\arg\min_{X_{\cdot ,\ell }}{\mathrm{TV}}_{2D}\left(X_{\cdot ,\ell }\right)\;\;\;\mathrm{subject}\mathrm{to}Y_{\cdot ,\ell }=\Phi X_{\cdot ,\ell }$$

#### 2.2.3. Multitrack Adaptive Compressed Sensing

This section describes the second of our proposed methods. It involves implementing an adaptive basis scan algorithm where multiple basis coefficients are sampled in parallel. Preliminary results were published in [43]. Similar to most adaptive basis scans, we use a 2D wavelet basis—specifically, 2D Haar wavelets [37]. Besides their simplicity, Haar wavelets have a property that is critical to our parallelized sampling: when viewed as a $n_{h}$-by-$n_{w}$ pixel 2D pattern, the support (i.e., region of non-zero pixels) of a Haar wavelet is localized and can be completely contained inside a track or subpattern. This allows for sampling a wavelet coefficient using only a subpattern instead of a full $n_{h}$-by-$n_{w}$ pattern. The other subpatterns are free to parallelly sample other wavelet coefficients. Details of the process are described below.

#### Measurement of Wavelet Coefficients

This problem consists of designing *compound patterns* made up of the subpatterns, such that each of the recorded spectra provide information on the wavelet coefficients. Consider a 2D Haar wavelet $\psi_{q}\in R^{n_{p}}$ labeled by an index *q*. This, when viewed as a a $n_{h}$-by-$n_{w}$ 2D wavelet pattern, can also be divided into $n_{\tau }$ tracks of size $n_{h}$-by-$\left(n_{w}/n_{\tau }\right)$, where the division into tracks can again be described by breaking down $\psi_{q}$ into submatrices:

(15) $$\psi_{q}=\left[\begin{array}{c} \psi_{q}^{\left(1\right)}\\ \psi_{q}^{\left(2\right)}\\ \vdots \\ \psi_{q}^{\left(n_{\tau }\right)} \end{array}\right]$$

Here, $\psi_{q}^{\left(i\right)}\in R^{\left(n_{p}/n_{\tau }\right)}$ is the *i*th track of the wavelet. Now, if $\psi_{q}$ is a Haar wavelet pattern whose support lies entirely in the *i*th track, then its pixel values must be zero in all other tracks. This would mean that for any track $i^{\prime }$ other than *i*, we have $\psi_{q}^{\left(i^{\prime }\right)}=0$, and $\psi_{q}$ reduces to:

(16) $$\psi_{q}=\left[\begin{array}{c} 0\\ \vdots \\ \psi_{q}^{\left(i\right)}\\ \vdots \\ 0 \end{array}\right]$$

Hence, the sub-matrix (or subpattern) $\psi_{q}^{\left(i\right)}$ can play the role of the full wavelet $\psi_{q}$, despite being smaller in size. Importantly, it can still sample dot products:

(17) $$\psi_{q}^{T}X=\left[\begin{array}{ccccc} 0^{T} & \cdots & {\left(\psi_{q}^{\left(i\right)}\right)}^{T} & \cdots & 0^{T} \end{array}\right]\left[\begin{array}{c} X^{\left(1\right)}\\ \vdots \\ X^{\left(i\right)}\\ \vdots \\ X^{\left(n_{\tau }\right)} \end{array}\right]={\left(\psi_{q}^{\left(i\right)}\right)}^{T}X^{\left(i\right)}$$

Note that the left-hand side is just the wavelet coefficients of the slices of *X*: $\psi_{q}^{T}X=\left[\psi_{q}^{T}X_{\cdot ,1},\ldots ,\psi_{q}^{T}X_{\cdot ,n_{c}}\right]=\left[W_{q,1},\ldots ,W_{q,n_{c}}\right]$. Thus, wavelet subpatterns can be used instead of full wavelets to obtain the wavelet coefficients. This, together with the aforementioned facts that (1) several DMD subpatterns $\zeta^{\left(i\right)}$ can be formed parallelly on the DMD and (2) the corresponding spectra can be measured, enables the extraction of multiple wavelet coefficients per slice in a shot.

The precise steps involved in this process are as follows. Let $W_{q_{1},\ell },W_{q_{2},\ell },\ldots ,W_{q_{n_{\tau }},\ell }$, for all channels $\ell \in \{1,\ldots ,n_{c}\}$, be wavelet coefficients that are to be extracted simultaneously in a shot. To enable this, we require that the supports of the corresponding patterns $\psi_{q_{1}},\psi_{q_{2}},\ldots ,\psi_{q_{n_{\tau }}}$ lie in separate tracks. Without the loss of generality, suppose the support of $\psi_{q_{i}}$ lies in the *i*th track for each $i\in \{1,\ldots ,n_{\tau }\}$. Then, from (17),

(18) $$\left[W_{q_{i},1},\ldots ,W_{q_{i},n_{c}}\right]=\psi_{q_{i}}^{T}X={\left(\psi_{q_{i}}^{\left(i\right)}\right)}^{T}X^{\left(i\right)}$$

Using this equation, we can obtain a formula similar to Equation (11) that extracts the wavelet coefficients from the measured raw spectra. Analogous to Section 2.2.2, we employ the following scaling and shifting operations on the wavelet subpatterns to account for the non-negative and discretized range of the DMD pixel values:

(19) $$\zeta_{q_{i}}^{\left(i\right)}=\frac{2^{b-1}-1}{2^{b}-1}\left(\frac{\psi_{q_{i}}^{\left(i\right)}}{{\left\|\psi_{q_{i}}^{\left(i\right)}\right\|}_{\infty }}+1_{n_{p}}^{\left(i\right)}\right)\;\;\;\;\mathrm{for}\mathrm{all}\mathrm{tracks}i$$

where *b* is the number of bits again, and $1_{n_{p}}^{\left(i\right)}$ is a subpattern ${\left[1,\ldots ,1\right]}^{T}\in R^{\left(n_{p}/n_{\tau }\right)}$ of the all-ones pattern $1_{n_{p}}$. Again, an additional set of offset subpatterns is required to account for the scaling and shifting mentioned above:

(20) $$\zeta_{\mathrm{offset}}^{\left(i\right)}=\frac{2^{b-1}-1}{2^{b}-1}\cdot 1_{n_{p}}^{\left(i\right)}\;\;\;\;\mathrm{for}\mathrm{all}\mathrm{tracks}i$$

Now, the final subpatterns $\zeta_{q_{i}}^{\left(i\right)}$ for $i\in \{1,\ldots ,n_{\tau }\}$ can be formed parallelly as a compound pattern on the DMD (Figure 1b). The pixels on the *i*th track of the DMD form the *i*th subpattern. With the integration time Δt, the recorded spectra (see Equation (4)) are:

(21) $$G\left(q_{1},\ldots ,q_{n_{\tau }}\right)=\left[\begin{array}{c} {\left(\zeta_{q_{1}}^{\left(1\right)}\right)}^{T}X^{\left(1\right)}\Delta t\\ \vdots \\ {\left(\zeta_{q_{n_{\tau }}}^{\left(n_{\tau }\right)}\right)}^{T}X^{\left(n_{\tau }\right)}\Delta t \end{array}\right]$$

A set of measurements from the offset subpatterns is also needed:

(22) $$G_{\mathrm{offset}}=\left[\begin{array}{c} {\left(\zeta_{\mathrm{offset}}^{\left(1\right)}\right)}^{T}X^{\left(1\right)}\Delta t\\ \vdots \\ {\left(\zeta_{\mathrm{offset}}^{\left(n_{\tau }\right)}\right)}^{T}X^{\left(n_{\tau }\right)}\Delta t \end{array}\right]$$

Denoting the (i,ℓ)th entries of $G(q_{1},\ldots ,q_{n_{\tau }})$ and $G_{\mathrm{offset}}$ by ${\left[G\left(q_{1},\ldots ,q_{n_{\tau }}\right)\right]}_{i,\ell }$ and ${\left[G_{\mathrm{offset}}\right]}_{i,\ell }$, respectively, we finally obtain as a result of Equation (18):

(23) $$W_{q_{i},\ell }=\frac{2^{b}-1}{2^{b-1}-1}\frac{{\left\|\psi_{q_{i}}\right\|}_{\infty }}{\Delta t}\left({\left[G\left(q_{1},\ldots ,q_{n_{\tau }}\right)\right]}_{i,\ell }-{\left[G_{\mathrm{offset}}\right]}_{i,\ell }\right)\;\;\;\;\mathrm{for}\mathrm{all}i,\ell$$

#### Extension of the Adaptive Basis Scan Algorithm to Incorporate Multitrack Measurements

**Figure 2:**
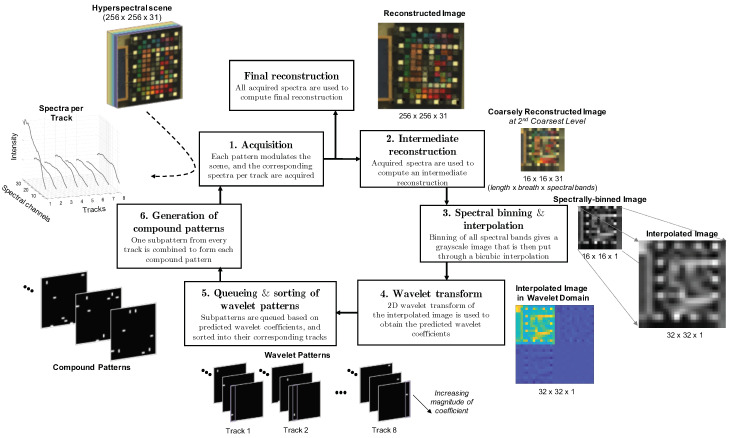
A flow chart of the multitrack adaptive CS scheme for hyperspectral imaging. Steps 1–6 are described briefly here.

Steps 1–4 in Figure 2 are closely based on Rousset et al. [29,39] and will be described only briefly here. Steps 5 and 6 are novel and designed to incorporate the multitrack requirement into the adaptive algorithm.

The setup is similar to [29,39]. The decomposition level of the wavelet transform is set to some J∈N, which determines the coarsest scale at which information from the scene is captured. However, the need for multitrack sampling imposes a restriction on *J* due to the requirement that the support of each wavelet pattern must be situated completely within the boundaries of a track. Since the support of a Haar wavelet under decomposition level *J* can be up to $2^{J}$-by-$2^{J}$ pixels in size, we need $2^{J}$ to be less than the width of a track in pixels: $2^{J}\leq n_{w}/n_{\tau }$.

Next, the sampling percentages are set for each scale $p_{J},\ldots ,p_{1}$, with j=J being the coarsest, and j=1 being the finest.

The algorithm is divided into J+1 acquisition cycles where wavelet coefficients are measured, and *J* prediction cycles where the significance of yet-to-be-measured wavelet coefficients is predicted using the already-measured coefficients. The resultingpredicted wavelet coefficients then guide the next acquisition cycle and so on.

The algorithm starts off with an acquisition cycle (**Step 1** in Figure 2) where *all* the *approximation wavelet coefficients* of all slices $X_{\cdot ,\ell }$ of the scene are measured. Unlike [29,39], these are now measured parallelly with the multitrack system using the corresponding approximation compound patterns made up of subpatterns that extract approximation coefficients. The first prediction cycle (described in the next paragraph) predicts the *detail wavelet coefficients* at scale j=J, and this is followed by an acquisition cycle that measures detail coefficients at scale j=J. The scale is then lowered by one, j←j−1, and the cycles repeat.

In a prediction cycle for detail coefficients at scale j∈{1,…,J}, the steps are as follows. First, a low-resolution intermediate reconstruction is obtained by inverse transforming all the wavelet coefficients that were measured or set to zero in all the previous acquisition cycles for every slice (**Step 2** in Figure 2). Binning of all slices at the various wavelength channels gives a grayscale image, which is then oversampled by a factor of 4 in the number of pixels via a bicubic interpolation. (**Step 3** in Figure 2). The oversampled image is then one-level 2D wavelet transformed to give predicted detail wavelet coefficients at scale *j* (**Step 4** in Figure 2). Until now, all the prediction steps have been similar to the ABS-WP algorithm from [29,39]. The next two steps contain the differences that are needed to make ABS-WP compatible with the multitrack measurement (see also **Steps 5** and **6** in Figure 2).

**Step 5**: Predicted wavelet coefficients are queued according to their magnitudes. The higher the magnitude of a predicted wavelet coefficient, the earlier in the queue it appears, as it is expected to be more significant. Each predicted wavelet coefficient will correspond to a unique wavelet, creating an ordered queue of wavelets. Viewing the wavelets as 2D patterns, the support of each wavelet pattern is completely situated within the boundaries of a track, and the corresponding track that the support sits within can be referred to as the pattern’s supporting track. The queued wavelet patterns are further sorted into sub-queues based on their supporting tracks while maintaining their ordering within each sub-queue. Based on $p_{j}$, a corresponding percentage of the more significant wavelet patterns is retained from each of the sub-queues, while the less significant wavelet patterns (at the back of the queues) are discarded.

However, this sub-queuing process can result in some imaging accuracy losses. In the single-track ABS-WP, the patterns to be used correspond to the $p_{j}$ most significant predicted coefficients overall. This is different, in general, however, from the $p_{j}$ most significant coefficients from each sub-queue because some sub-queues might have more of the significant coefficients than other sub-queues.

**Step 6**: One wavelet pattern from each sub-queue is combined to form a compound pattern: if the wavelet patterns $\psi_{q_{1}},\psi_{q_{2}},\ldots ,\psi_{q_{n_{\tau }}}$ appear first in sub-queues $1,2,\ldots ,n_{\tau }$, respectively, (so that the supporting track of $\psi_{q_{i}}$ is the *i*th track), then the first compound pattern is formed by corresponding subpatterns $\zeta_{q_{i}}^{\left(i\right)}$, for $i\in \{1,\ldots ,n_{\tau }\}$, obtained from Equation (19). This is continued sequentially, with the second wavelet pattern from each sub-queue combined into the second compound pattern and so on.

This prediction cycle at scale *j* is followed by an acquisition cycle, as mentioned before, for the measurement of detail coefficients at scale *j* for all slices $X_{\cdot ,\ell }$ of the scene. The compound patterns from **Step 6** are used to parallelly measure the detail coefficients that were predicted to be significant in the preceding prediction cycle. The rest of the coefficients at scale *j* are set to zero. Finally, *j* is lowered by one and the cycles repeat. The algorithm terminates when j=0.

The final reconstruction is obtained by an inverse wavelet transform, for each slice, of all the wavelet coefficients that were measured (or set to zero) from that slice of the scene in all the acquisition cycles. This is identical to the reconstruction step in [39].

## 3. Results and Discussion

### 3.1. Numerical Experiments

All numerical experiments/simulations in this work are conducted using MATLAB 2018a on a HP Z240 Tower Workstation with 4 cores of Intel® Core™ i5-6500 CPU @ 3.20 GHz and 16 GB of RAM.

The performance and applicability of multitrack CS for HSI is assessed computationally on hyperspectral images. The measurements are simulated and fed into the adaptive sampling or reconstruction algorithms being evaluated.

More precisely, a ground truth hyperspectral datacube is treated as the scene to be imaged *X*. Simulated measurements are computed according to the forward model from Equation (4), where the corresponding subpatterns $\zeta_{k}^{\left(i\right)}$ are generated according to the reconstruction algorithms to be used. For multitrack non-adaptive CS, the subpatterns are obtained from Equation (7), whereas for multitrack adaptive CS, the ABS-WP algorithm and Equation (19) determine the subpatterns. These computations yield the simulated measurements *G* (see Equations (9) and (21)), which are used to reconstruct the hyperspectral image. The reconstructions are then compared against the ground truth images; the details of these comparisons are provided later in this section.

First, noiseless measurement simulations and reconstruction are performed on three different hyperspectral images: Color Checker, Green Peppers, and U2OS cell, obtained from [44,45]. The former two are 512-by-512-by-31 voxel macroscopic images; the latter is a 256-by-256-by-231 voxel coherent anti-Stokes Raman Scattering (CARS) hyperspectral image. Both multitrack non-adaptive CS (Section 2.2.2) and multitrack adaptive CS (Section 2.2.3) are compared against benchmarks:

Multitrack full sampling: This involves sampling the whole datacube using a scheme similar to pushbroom. However, for a fair comparison with the multitrack CS methods, it is equipped with the same acquisition architecture as the multitrack methods—the same number of detector tracks, in particular. All spectra are measured from each track of the scene in a ‘multitrack point-scan’ fashion. Hence, no reconstruction step is needed.

Single-track adaptive CS: This involves the sampling of a single spectrum per shot by a SPC-style hyperspectral imager [15], using the adaptive ABS-WP algorithm of [29]. Note that we use an adaptive algorithm for benchmarking here because it generally outperforms single-track non-adaptive CS in terms of speed as well as accuracy.

Details of the compared methods are summarized in Table 1. For multitrack non-adaptive CS, the sparse recovery algorithm used is total variation (TV) minimization with equality constraint, implemented using the TVAL3 package [46]. The settings used are ‘anisotropic TV’ with ‘positivity’ since these tend to give the best results. For multitrack adaptive CS as well as single-track adaptive CS, the decomposition level used is J=4 for Color Checker and Green Peppers, and J=3 for U2OS cell. Higher values of *J* enable the use of smaller sampling ratios and are generally preferable. However, since the U2OS cell scene is of a smaller pixel count, our use of multitrack adaptive CS requires *J* to be less than four to ensure that the supports of the wavelet patterns lie completely inside individual tracks.

The results are reported in Section 3.2. The performance of the compared methods is quantified by the following metrics:

Reconstruction accuracy: This is quantified by relative error and peak signal-to-noise ratio (PSNR).

Acquisition time: This is composed of (i) the time required to adaptively determine the DMD patterns (0 for non-adaptive), (ii) the time required to load the DMD patterns into a computer’s memory, (iii) the time required to switch the DMD patterns, as per the pattern switch rate, and (iv) the total spectrometer integration time, which is the total number of shots times Δt.

Reconstruction time: This is the time required to compute the final reconstruction.

Total time: Acquisition time + Reconstruction time.

In Section 3.3, we report the simulated effects of the illumination level and noise on imaging accuracy and assess the robustness of the methods developed here. We model measurement noise as Poissonian shot noise together with dark noise. Given a subpattern $\zeta^{\left(i\right)}$ for track *i* and integration time Δt, the recorded noisy spectrum from the track is modeled as

(24) $$\mathrm{Pois}\left({\left(\zeta^{\left(i\right)}\right)}^{T}X^{\left(i\right)}\Delta t\;+\;\alpha \Delta t\right)$$

where $\alpha \in R_{+}$ is the dark current in the detector pixels in units of photons pixel${}^{-1}$$s^{-1}$, and Pois(μ) stands for a Poisson random variable with mean μ.

In low-light imaging, where the scene intensity is low, both shot noise and dark noise become more prominent.

Shot noise: The standard deviation of a Pois(μ) random variable is $\sqrt{\mu }$; as a result, the ratio (coefficient of variation) $\frac{\sqrt{\mu }}{\mu }$ grows larger as μ shrinks. The implication is that when the intensity of the scene *X* is small, the standard deviation of the measured signal is large with respect to its mean.

Dark noise: The αΔt term can overpower the ${\left(\zeta^{\left(i\right)}\right)}^{T}X^{\left(i\right)}\Delta t$ term when the scene intensity is small.

### 3.2. Simulation Results on Various Hyperspectral Images

**Figure 3:**
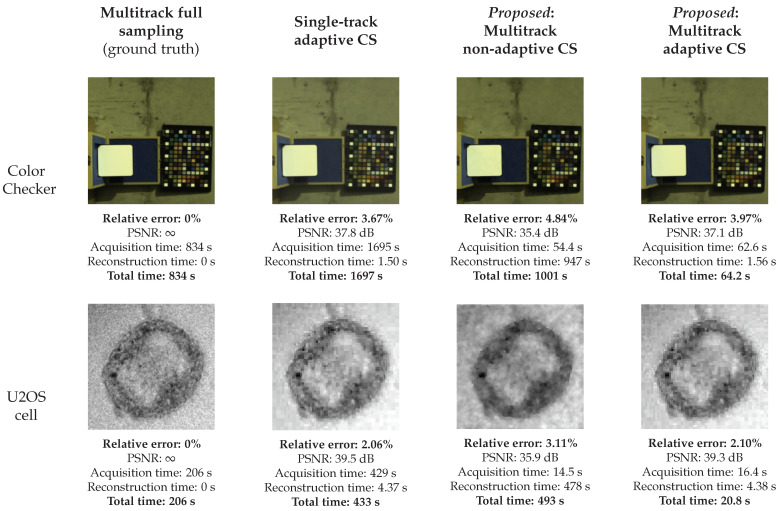
Simulations are carried out on the hyperspectral scenes Color Checker and U2OS cell. The reconstruction results are presented for each of the methods tested. For the Color Checker scene, pseudo-RGB versions of the reconstructed images are displayed in the table. For the U2OS cell scene, the 2964.5 ${\mathrm{cm}}^{-1}$ band of the reconstructed image is displayed.

The simulation results for the Color Checker and U2OS cell images under noiseless measurement are presented in Figure 3. We evaluate the two proposed multitrack methods against multitrack full sampling and single-track adaptive CS. First, we observe that single-track acquisition is slow: its acquisition time (and total time) is about twice that of multitrack full sampling. Since only one spectrum is measured per shot, a large number of shots are required even with a low sampling ratio of 6.2%. The other three methods, on the other hand, need fewer shots due to their multitrack acquisition architecture.

Multitrack non-adaptive CS had the shortest acquisition time—about 6–7% of the acquisition time of full sampling enabled by the employed sampling ratio of 6.2%. For the 512-by-512-by-31 voxel Color Checker scene, full sampling required 8192 shots, whereas sampling at 6.2% amounted to 516 shots. Note that the 516 shots here include 10 extra shots corresponding to the offset patterns mentioned in Section 2.2. A similar comparison applies to the U2OS cell scene, which is 256-by-256-by-231 voxels. A low sampling ratio, together with multitrack acquisition, reduced the acquisition time significantly. Furthermore, no adaptive prediction steps were needed during the acquisition process, unlike for multitrack adaptive CS. Hence, the pattern sequence is not computed on the fly and can be pre-programmed into the DMD’s field-programmable gate array (FPGA) for increased speed. However, due to long reconstruction times (947 s for the Color Checker scene and 478 s for the U2OS cell scene), the total time for multitrack non-adaptive CS is even longer than that of full sampling. Thus, despite having a fast acquisition step, the long reconstruction time necessitates offline post-processing.

In terms of total time, multitrack adaptive CS performs the best. It is faster than multitrack non-adaptive CS due to a much shorter reconstruction time, even though the acquisition time is up to 8 s higher due to the adaptive steps. Multitrack adaptive CS is also faster than full sampling, again, because of the small sampling ratio that required fewer shots.

For the multitrack methods, an increase in the number of tracks improves the imaging speed due to a higher extent of parallelization in sampling. However, this is valid only up to a limit in the case of multitrack adaptive CS—the tracks still have to be wide enough to contain the supports of the wavelet patterns, as was discussed earlier.

Regarding reconstruction accuracy, the simulation results show that multitrack adaptive CS outperforms its non-adaptive counterpart for both the Color Checker and the U2OS cell scene; the PSNR for multitrack adaptive CS is 2–3 dB higher. Hence, there is evidence that the wavelet coefficients measured capture the important information from the scene effectively. This discrepancy in accuracy could also be due to the scenes being more easily representable in the wavelet basis or due to the scenes not having sufficiently low total variation as would be necessary for TV-based sparse recovery. Full sampling achieves the best imaging accuracy; however, the (relative) errors are smaller than 5% for the multitrack CS methods. The slight loss of accuracy for a huge gain in speed presents a good tradeoff in favor of multitrack adaptive CS in particular.

Besides the above comparisons, one needs to make sure that the accuracy of multitrack adaptive CS is not much behind that of single-track adaptive CS. The simulation results indeed confirm that despite the losses induced by the sub-queue formation in multitrack adaptive CS, the PSNR discrepancy of the two methods does not exceed 1 dB. Hence, this shows that the ABS-WP algorithm has been successfully extended to the multitrack measurement scenario.

**Figure 4:**
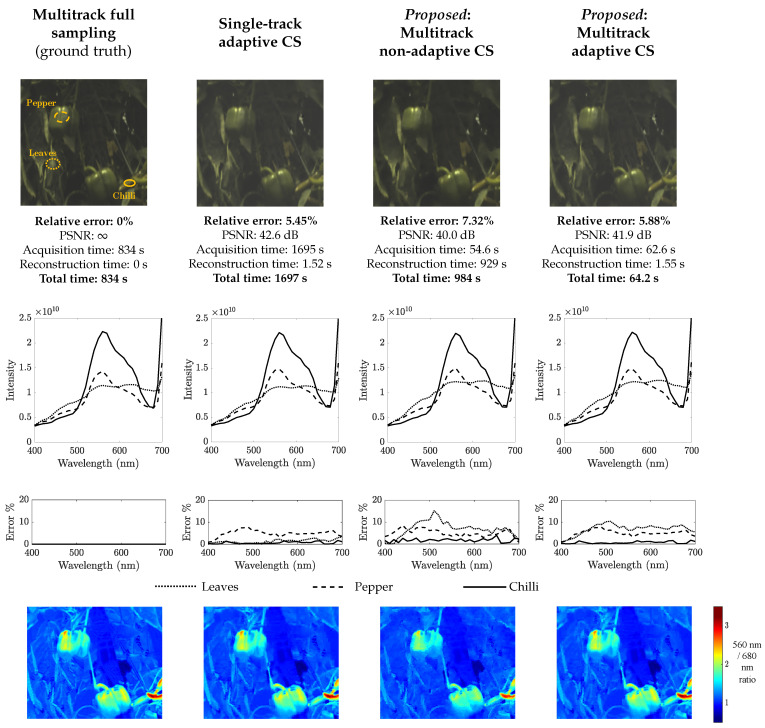
Simulations are carried out on the hyperspectral scene Green Peppers. The reconstruction results are presented for each of the methods tested. Pseudo-RGB versions of the reconstructed images are displayed in the first row. Reconstructed spectra are plotted in the second row. The dotted lines (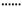) are from a point on the green leaves background; the dashed lines (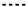) are from a point on the pepper near the top left corner; and the solid lines (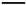) are from a point on a chilli at the bottom right corner, as indicated on the ground truth image. The third row shows the errors of the reconstructed spectra relative to the ground truth spectra. Finally, the fourth row displays the pixel-wise ratio of the spectral bands at 560 and 680 nm.

Next, Figure 4 shows a similar comparison between the four methods for the Green Peppers image. The first row of Figure 4 yields similar conclusions as Figure 3: multitrack non-adaptive CS has the shortest acquisition time; multitrack adaptive CS has the shortest total time; and the relative errors of the multitrack CS methods, though higher than in Figure 3, are still only about 6–7%. In the second, third and fourth rows of Figure 4, we demonstrate the potential applicability of multitrack CS HSI to agricultural monitoring. Reconstructed spectra from each of the compared methods are plotted in the second row. The dotted lines are from a point on the green leaves background; the dashed lines are from a point on the pepper near the top left corner; and the solid lines are from a point on a chili at the bottom right corner, as indicated on the ground truth image. The multitrack CS methods can distinguish the three, as can full sampling and single-track. The third row shows the errors of the reconstructed spectra from the various methods relative to the ground truth spectra. The errors are low for the multitrack CS methods and do not prevent us from distinguishing the different regions while still being fast. The exact trends of the error curves likely depend on the shape of the spectra and the intensity levels. Finally, the fourth row displays the pixel-wise ratio of the spectral bands at 560 and 680 nm. The ratio images clearly distinguish the peppers from the background for each of the compared methods, possibly making it easier to perform semantic segmentation. Note that the red regions at the bottom right corners correspond to chilies.

### 3.3. The Effect of Illumination Level and Noise

We modeled measurement noise as Poissonian shot noise with dark noise, as described in Section 3.1. The factors determining the severity of noise were intensity of the scene *X*, integration time Δt and dark current α. The effects of these factors on the CS reconstructions of the U2OS cell scene are examined here via simulations for the multitrack methods. We do not analyze single-track CS further since we established in Section that it is much slower than the multitrack methods.

First, we set α=0 and note that *X* and Δt play a similar role in the resulting measurement:

$$\mathrm{Pois}\left({\left(\zeta^{\left(i\right)}\right)}^{T}X^{\left(i\right)}\Delta t\right)$$

Hence, it is reasonable to evaluate the effect of the product XΔt on the reconstruction accuracy instead of using *X* and Δt individually. Furthermore, XΔt has units of photons and, therefore, is physically meaningful as the photon counts emanating from the scene during the integration time interval. As a result, we quantify the illumination level by max(XΔt)—the max photon count per voxel emanating from the scene during the time interval Δt.

**Figure 5:**
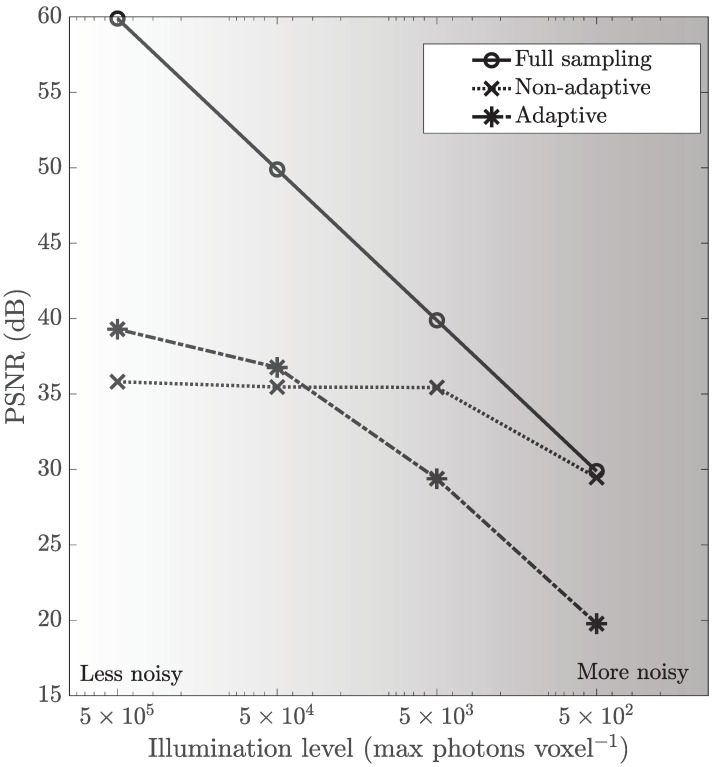
The effect of the illumination level on the reconstructed image PSNR of the U2OS cell scene for multitrack full sampling, multitrack non-adaptive CS and multitrack adaptive CS. The illumination level (horizontal axis) decreases from left to right, corresponding to an increasing prominence of shot noise. The gray shading illustrates the illumination level. The dark current α is fixed at 0.

Figure 5 shows the simulation results—the effect of illumination level on the reconstructed image accuracy (in PSNR)—for multitrack full sampling, multitrack non-adaptive CS and multitrack adaptive CS. The illumination level plotted on the horizontal axis decreases from left to right, corresponding to an increasing prominence of shot noise.

The reconstruction accuracy is highest for full sampling, similar to Section 3.2, across all illumination levels. However, the accuracy of non-adaptive CS starts approaching that of full sampling at lower illumination levels of ∼5 $\times 10^{2}$ photons voxel${}^{-1}$. We also note that for illumination levels higher than ∼5 $\times 10^{4}$ photons voxel${}^{-1}$, the accuracy of adaptive CS overtakes that of non-adaptive CS.

A prominent trend is that the reconstruction accuracies of full sampling and adaptive CS are more sensitive to the illumination level as compared to non-adaptive CS. The DMD patterns used in the former two have small supports, such that only a small percentage of incident light is reflected towards the detector. This leads to a weaker signal at the detector, which is more susceptible to shot noise. On the contrary, the DMD patterns used in non-adaptive CS reflect about 50% of the incident light towards the detector, which is much higher than the other two methods. The resultant stronger signal at the detector is thus less affected by shot noise.

Noting that this sensitivity is a measure of robustness to shot noise, we can conclude from comparing the slopes of the PSNR-illumination plots that the robustness of our CS methods is better than the robustness of full sampling.

**Figure 6:**
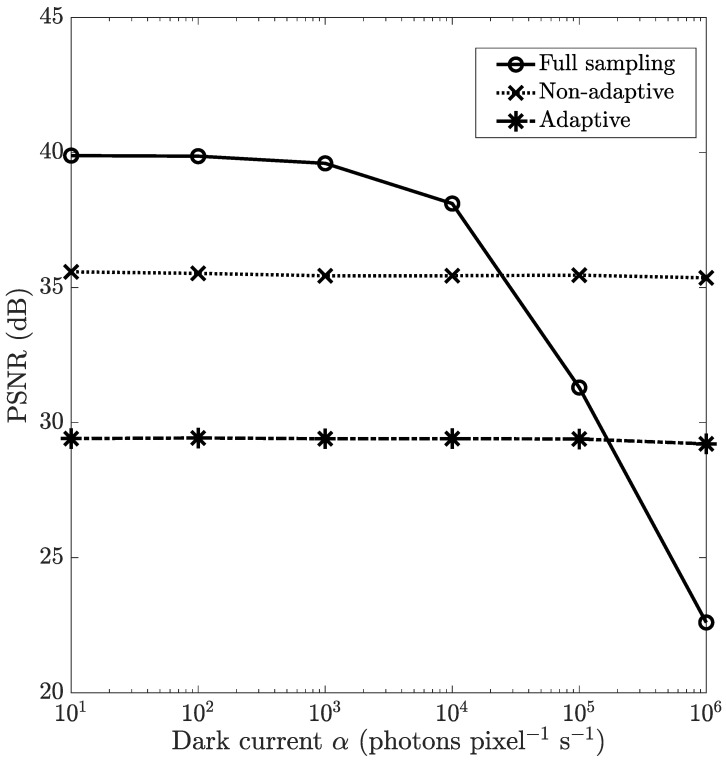
The effect of dark current α on the reconstructed image PSNR of the U2OS cell scene for multitrack full sampling, multitrack non-adaptive CS and multitrack adaptive CS. The max scene intensity is fixed at $5\times 10^{4}$ photons s${}^{-1}$ voxel${}^{-1}$, and the integration time is 0.1 s.

Next, we evaluate the effect of the dark current by varying α. We fix the max scene intensity at $5\times 10^{4}$ photons s${}^{-1}$ voxel${}^{-1}$ and the integration time 0.1 s, yielding an illumination level of $5\times 10^{3}$ photons voxel${}^{-1}$. Figure 6 shows the simulation results—the reconstruction accuracy across a wide range of dark current values. The plots indicate that full sampling is much more sensitive to dark current as compared to adaptive and non-adaptive CS. This gives the CS methods an advantage—their robustness to dark noise can allow the use of cheaper detectors or relax the cooling requirements of detectors.

These discrepancies could again be attributed to signal strengths at the detector, as mentioned earlier. Full sampling uses delta function subpatterns, which have smaller supports than the wavelet patterns used in adaptive CS and the random patterns used in non-adaptive CS. Furthermore, the CS methods have a regularization method built into them—sparsity in the wavelet domain for adaptive CS, and low TV norms for non-adaptive CS—making them more noise-robust in general.

**Figure 7:**
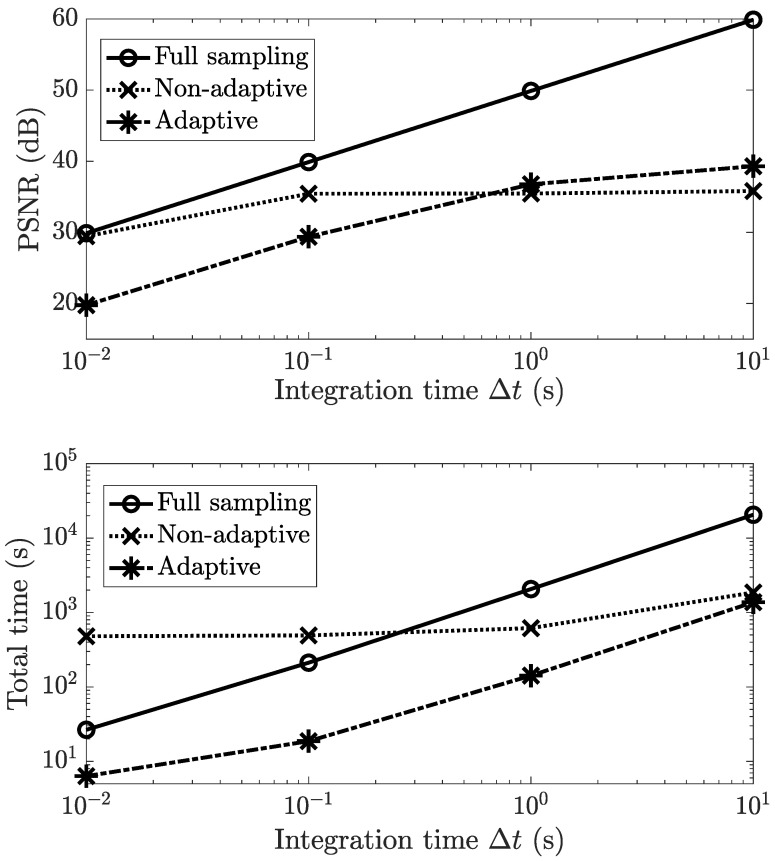
The effect of the integration time Δt on the U2OS cell scene’s reconstruction PSNR and total time (acquisition + reconstruction) for multitrack full sampling, multitrack non-adaptive CS and multitrack adaptive CS. α=0 and $\max\left(X\right)=5\times 10^{4}$ photons s${}^{-1}$ voxel${}^{-1}$ are fixed.

Lastly, we examine the effect of the integration time. Both reconstruction accuracy and acquisition time are affected by this quantity, leading to tradeoffs. The effect of the integration time on the accuracy was effectively captured in Figure 5 since the illumination level was determined by XΔt, which is linear in Δt. The Δt–PSNR relationship is plotted explicitly in the top panel of Figure 7 for the multitrack methods. Here, we have fixed α=0 and $\max\left(X\right)=5\times 10^{4}$ photons s${}^{-1}$ voxel${}^{-1}$.

The acquisition time and total time are related to the integration time as per the following equation:

(25) $$\begin{array}{l} \mathrm{total}\mathrm{time}=(\mathrm{number}\mathrm{of}\mathrm{shots}\times \Delta t)\\ \;+\mathrm{DMD}-\mathrm{pattern}-\mathrm{related}\mathrm{time}+\mathrm{reconstruction}\mathrm{time} \end{array}$$

The DMD-pattern-related time is independent of the integration time and comprises of (i) the time required to adaptively determine the DMD patterns (0 s for non-adaptive), (ii) the time required to load the DMD patterns into a computer’s memory and (iii) the time required to switch the DMD patterns, as per the pattern switch rate. Similarly, the reconstruction time is also independent of the integration time. These components are offset terms in Equation (25). The remaining term provides a linear relationship between the total time and Δt, the slope of which is governed by the sampling ratio. The bottom panel of Figure 7 plots these relationships for the three multitrack methods.

The total time curve for full sampling is the steepest due to a larger number of shots. Non-adaptive CS, on the other hand, has the highest reconstruction time, leading to a large offset when Δt→0. The lowest total time is achieved by adaptive CS, as was also shown in Section 3.2.

Both panels in Figure 7 can be used together for choosing the optimal multitrack method for the given accuracy and time requirements. Suppose a minimum PSNR must be achieved. Then the top panel would yield the integration times required by each of the methods to achieve this PSNR threshold. Based on the integration times, the bottom panel would then predict the total time required by the three methods, allowing us to choose the one with the smallest time. Conversely, if we have a maximum total time requirement, a similar procedure based on Figure 7 could then be used to choose the method that achieves the highest PSNR under this time constraint.

## 4. Conclusions

In this paper, we have developed two methods for multitrack compressed-sensing-based hyperspectral imaging—a non-adaptive approach based on sparse recovery and an adaptive approach based on sampling significant wavelet coefficients of the hyperspectral scene.

We have verified via simulations that both the methods can achieve short measurement times while maintaining high imaging accuracy. However, they have different advantages: the adaptive method enjoys a much shorter reconstruction time and computational burden, whereas the non-adaptive method is more robust to noise.

Next, we have also shown that the two methods are generalizable to different types of scenes—the reflectance of macroscopic objects as well as Raman signals of cells. This is likely because the methods do not rely upon information from fixed data sets.

Overall, the CS-HSI methods achieve a reconstruction accuracy close to full sampling, and the noise-robustness is even better than full sampling. This, along with the high acquisition speed as well as high reconstruction speed in the case of multitrack adaptive CS, demonstrates these methods to be effective.

## Figures and Tables

**Table 1:** A summary of methods and a comparison of their key features.

	Multitrack Full Sampling	Single-Track Adaptive CS	Multitrack Non-Adaptive CS	Multitrack Adaptive CS
Tracks:	32 tracks	1 track	32 tracks	32 tracks
Sampling ratio:	100%	About 6.2%	About 6.2%	About 6.2%
Integration time:	0.1 s per shot	0.1 s per shot	0.1 s per shot	0.1 s per shot
DMD pattern switch rate:	10,638 frames/s (1-bit binary patterns)	255 frames/s (8-bit grayscale patterns)	255 frames/s (8-bit grayscale patterns)	255 frames/s (8-bit grayscale patterns)

## Data Availability

The hyperspectral image data used in this paper was obtained from the public database http://icvl.cs.bgu.ac.il/hyperspectral/, accessed on 20 November 2019. and (upon request) from http://dx.doi.org/10.17035/d.2015.100098, accessed on 10 October 2019.

## Abbreviations

The following abbreviations are used in this manuscript:

HSI	Hyperspectral imaging
CS	Compressed sensing
CASSI	Coded aperture snapshot spectral imaging
CS-HSI	Compressed sensing based hyperspectral imaging
SPC	Single pixel camera
DMD	Digital micromirror device
ABS-WP	Adaptive basis scan with wavelet prediction
TV	Total variation
CARS	Coherent anti-Stokes Raman Scattering
PSNR	Peak signal-to-noise ratio
RGB	Red Green Blue
FPGA	Field-programmable gate array
